# A Recombinant Enolase-Montanide™ PetGel A Vaccine Promotes a Protective Th1 Immune Response against a Highly Virulent *Sporothrix schenckii* by Toluene Exposure

**DOI:** 10.3390/pharmaceutics11030144

**Published:** 2019-03-25

**Authors:** Damiana Téllez-Martínez, Deivys Leandro Portuondo, Maria Luiza Loesch, Alexander Batista-Duharte, Iracilda Zeppone Carlos

**Affiliations:** Department of Clinical Analysis, School of Pharmaceutical Sciences, São Paulo State University (UNESP), Araraquara 14800-903, SP, Brazil; damianatellezm@gmail.com (D.T.-M.); deivysleandro@gmail.com (D.L.P.); ma_luizaloesch@hotmail.com (M.L.L.)

**Keywords:** vaccine, adjuvants, *Sporothrix schenckii*, toluene, virulence, enolase, Montanide PetGel A

## Abstract

The effect of vaccination in fungal strains that suffered changes in their virulence by exposure to environmental contaminants is largely known. Growing reports of resistance to antifungal drugs and the emergence of new highly virulent strains, possibly acquired in the environment, prompt the design of new vaccines able to prevent and combat emerging mycotic diseases. In this study, we evaluated the protective capacity of an enolase-based vaccine and Montanide PetGel A (PGA) as an adjuvant against *S. schenckii* with increased virulence by exposure to toluene. The adjuvanted vaccine induced a strong specific Th1 response and protective immunity against a challenge with either wildtype or toluene-adapted *S. schenckii* in Balb/c mice. This study highlights the role of the adjuvant PGA driving the quality of the anti-sporothrix immunity and the key component in the vaccine efficacy.

## 1. Introduction

Sporotrichosis is an emergent subcutaneous mycosis in tropical and subtropical regions, caused by several pathogenic species of the genus *Sporothrix*; that include *Sporothrix brasiliensis*, *S. schenckii sensu stricto*, *Sporothrix globosa*, and *Sporothrix luriei* [1]. Classically, infection is acquired after traumatic inoculation of contaminated soil, plants, and organic matter into skin or mucosa or, more rarely, by inhalation of conidia. Over the last years, cat–human zoonotic transmission of sporotrichosis caused by *S. brasiliensis* has become a health problem in Brazil [2]. The disease can manifest as fixed cutaneous and regional lymphocutaneous forms in immunocompetent individuals, and disseminated forms, mainly reported in immunocompromised patients [3]. 

Ecological determinants of the genus *Sporothrix* remain poorly understood [4,5]. However, experimental evidence suggests that environmental contaminants can modify the fungal virulence by reducing the host immunity [6] or modifying the fungal biology [4,7]. Previous studies showed that fungal exposure to toluene, a common soil contaminant that shares the same environmental niche of *S. schenckii*, is able to increase the *S. schenckii* virulence [7]. Ongoing studies are evaluating the role of chemical contamination and other environmental factors in sporotrichosis outbreaks.

Conventional treatment of sporotrichosis requires long periods of antifungal drug administration often accompanied by adverse effects and fungal resistance, principally during the treatment of disseminated sporotrichosis [8]. These problems have stimulated the search for new strategies for sporotrichosis management, including anti-sporothrix vaccination that has been proposed as a feasible way for both therapeutic and prophylactic purposes [9,10]. However, the development of antifungal vaccines has not been as successful as antiviral and antibacterial vaccines due to, among other things, a general under-appreciation for the impact of fungal diseases and the high cost of preclinical and clinical studies [11]. Another challenge has been the use of immunological adjuvants with an adequate safety and efficacy profile [12]. Aluminium-derived adjuvants have been used in human and veterinary vaccines for more than eight decades in licensed vaccines [13,14]. However, they have not been successful in preventing intracellular infection due to a weak capacity to induce cell-mediated immunity when used along with small immunogenic antigens [13]. Moreover, there are reports of tumors in the inoculation site in felines vaccinated with alum-based vaccines and a possible causal association between chronic inflammation induced by alum and these tumors has been suggested [15]. 

Current advances in the understanding of antifungal immune response support rational use of more effective adjuvants, such as pattern recognition receptors (PRR) agonists, inhibitors of regulatory T cells, and others [12,16,17], to achieve effective immune responses against specific fungi.

**Figure 1:**
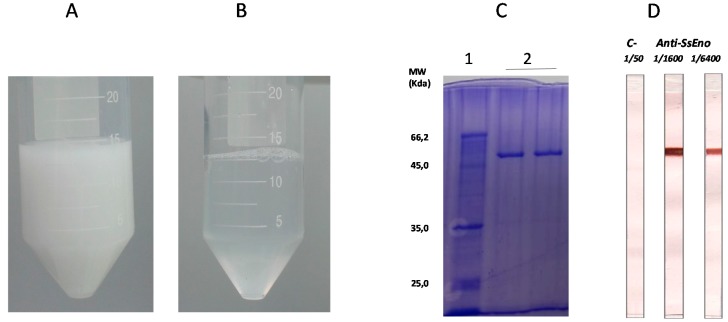
External aspect of (**A**) Montanide^TM^ PetGel (PGA) and (**B**) PGA+rSsEno vaccine formulation. (**C**) SDS-PAGE showing the expression and purification of *rSsEno.* C1. Molecular pattern, C2. rSsEno band stained with Coomassie blue with the expected 47 kDa molecular mass (in duplicate). (**D**) Immunoblotting analysis of rSsEno with a pooled anti-*rSsEno* serum from Balb/c mice immunized with Freund’s adjuvant/rSsEno (Either 1/1600 or 1/6400 dilutions of serum were used). A pooled serum from non-immunized mice (dilution 1/50) was used as negative control (C−) to identify non-specific binding.

For several decades, biodegradable natural and synthetic polymers have been used for antigen delivery and as immunological adjuvants. Due to their biocompatibility, biodegradability, easy production and low toxicity polymers are attractive candidates for substituting conventional adjuvants [18]. MontanideTM PetGel A (PGA), is a polymer-based adjuvant composed of highly stable dispersion of microspherical particles of sodium polyacrylate in water (Figure 1A, Table 1). This polymeric technology has already been used in several vaccine models, including pet vaccines, with a promising safety and efficacy profile [19].

Recently, our group evaluated a cell-wall protein extracted from *S. schenckii* (SsCWPs) in an experimental vaccine formulated with Aluminum hydroxide (AH) gel [20]. Immunized mice developed a specific immune response characterized by a balanced Th1/Th2/Th17 response and the production of protective antibody response. In a more recent study, the AH-based vaccine was compared with other experimental vaccine candidate containing SsCWPs and PGA. Both formulations induced a protective immune response in mice. However, AH stimulated the development of granulomas in the inoculation site while PGA-based vaccine exhibited a Th1 protective response and better local tolerance than AH-based vaccine in vaccinated mice [21]. In other recent study, we evaluated the immunogenic and protective effect of enolase, one of the main antigens that were found in SsCWPs, against *S. brasilensis*, the most virulent species of the genus *Sporothrix* [22]. The recombinant enolase of *S. schenckii* (SsEno) was formulated with PGA and after three subcutaneous administrations in mice, a significant specific immune response was observed. Furthermore, a reduction in mortality (over 90%) was observed after 45 days of an intravenous challenge with viable yeast, compared with non-vaccinated mice.

Until now, there are no studies on the effects of vaccination on fungal strains whose virulence changed due to exposure to environmental contaminants. In the current context of growing reports of fungal resistance to antifungal drugs and the emergence of new highly virulent strains, possibly acquired in the environment, these assessments can provide important information on the ability of new vaccines to prevent and combat emerging mycotic diseases.

In this study, we evaluated the protective capacity of an enolase-based vaccine formulated with PGA as an adjuvant, against *S. schenckii* with increased virulence by experimental exposure to toluene [7]. 

## 2. Materials and Methods

### 2.1. Animals

Male Balb/c mice (five to seven weeks old) were purchased from “Centro Multidisciplinar para Investigação Biológica na Área da Ciência de Animais de Laboratório” (CEMIB), Universidade de Campinas (UNICAMP), São Paulo, Brasil. Mice were housed in microisolator cages in a controlled ambient and receiving water and food ad libitum. The study was carried out in accordance with the Guide for the Care and Use of Laboratory Animals of the National Institutes of Health. The experiments were approved by the Ethics Committee for Animal Use in Research of Araraquara’s School of Pharmaceutical Sciences from UNESP (Protocol CEUA/FCF/CAr: 19/2018).

### 2.2. Microorganisms and Preparations

The *S. schenckii sensu stricto* strain ATCC 16345 (here named as *S. schenckii*) used in this work was kindly provided by the Oswaldo Cruz Foundation (Rio de Janeiro, Brazil). The mycelial phase was maintained at room temperature in Mycosel (BD Biosciences) agar. A piece of a well-defined colony was grown in 100 mL of Sabouraud dextrose broth (SDB) (Difco, Detroit, MI, USA) for four days in a rotary shaker (130 rpm and 30 °C). The conidia were separated from the hyphae by filtration with sterile gauze using a Buchner funnel. Them conidia were counted and suspended in phosphate-buffered saline (PBS) at 1 × 10^7^/mL. 

### 2.3. S. schenckii Growth in Toluene

Fungal cultures were performed in 125-mL Erlenmeyer flasks containing 50 mL of Sabouraud dextrose broth (SDB) and sealed with Teflon Mininert valves (SUPELCO, 24 mm, (Merck KGaA Darmstadt, Germany) to prevent evaporation of the solvent. SDB was supplemented with toluene 0.1% (*v*/*v*). An aliquot of 1 × 10^7^ conidia was inoculated and incubated during five days on a rotary shaker (30 °C and 130 rpm). Control cultures without toluene were included. Fungal viability was determined at fifth day by counting colony forming units (CFU) on Sabouraud dextrose agar (SDA) plates [7].

### 2.4. Expression and Purification of Recombinant S. schenckii Enolase (rSsEno)

The detailed procedures were previously described [22]. Briefly, the gene that encodes *S. schenckii* enolase with molecular mass 47 kDa and 438 amino acids (access code: ERS97971.1, GenBank database) was synthesized by Epoch Life Science Inc. (Missouri, TX, USA). The enolase gen was subcloned into the pET28a plasmid and optimized for production in *Escherichia coli* (pET28a::SsEno). *E. coli* DH5α was used for the propagation of pET28a::SsEno on lysogeny broth (LB) agar medium containing 30 μg/mL of kanamycin. For recombinant protein expression, *E. coli* BL21 cells cotransformed with pET28a::SsEno were grown at 37 °C in LB medium with kanamycin until they reached an OD600 in the range of 0.5–0.7. The expression of rSsEno was induced by 0.2 mmol/L of isopropyl β-D-1-thiogalactopyranoside (IPTG) at 30 °C for 4 h. The cells were centrifuged (20 min at 8000 rpm), and the pellet was resuspended in buffer A (NaPO4 20 mM, NaCl 500 mM and imidazole 20 mM, pH 7,4) containing 5 U of DNAse (Promega, Madison, WI, USA) and 30 µg/mL lysozyme (Merck KGaA Darmstadt, Germany) for 30 min on ice. The cell homogenate was sonicated, filtrated and then centrifuged at 19,000 rpm for 20 min at 4 °C. The supernatant containing rSsEno was filtered (0.45 µm nitrocellulose membrane, Millipore and initially purified by Ni^2+^-affinity chromatography in buffer A. The rSsEno eluted in buffer B (NaPO_4_ 20 mM, NaCl 500 mM, and imidazole 500 mM, pH 7.4) was subjected to size exclusion chromatography (SEC) with a Superdex 200 pg 16/60 column (GE Healthcare Life Sciences, Chicago, IL, USA) in Tris-HCl 25 mM, NaCl 100 mM and β-mercaptoethanol 2 mM at pH 7.5, and the eluted protein was concentrated using the Amicon^®^ Ultra 15 mL 3k device (Millipore, Burlington, MA, USA) after being dialyzed for 24 h at 4 °C against phosphate buffer saline. The rSsEno concentration was measured by the Pierce BCA assay (Thermo Scientific, Waltham, MA, USA), and the efficacy of the expression and purification processes was assessed by 12% SDS-polyacrylamide gel electrophoresis (SDS-PAGE) and immunoblotting using anti-rSsEno serum.

### 2.5. Adjuvants and Vaccine Formulation

The vaccine formulation was prepared by mixing 100 μg of rSsEno with 5% PGA adjuvant kindly provided by Seppic (Paris, France) (Figure 1B). Other formulations composed by either PGA or rSsEno alone were used as control. 

### 2.6. Immunization Schedule

Balb/c mice (*n* = 5) received subcutaneous (s.c.) vaccination (on days 0 for priming and 14 for booster) in the back of the neck, with 100 μL of one of the following formulations: PGA+rSsEno, 100 μg rSsEno or PBS alone as a negative control. One week after the booster, mice were euthanized in CO_2_ chamber and bled by heart puncture to obtain serum, which was aliquoted and stored at −20 °C until use.

### 2.7. Quantification of the rSsEno-Antibody Response by Enzyme-Linked Immunosorbent Assay (ELISA)

rSsEno IgG antibody titration was conducted as described previously [22]. Briefly, a 96-well ELISA plate (Merck KGaA Darmstadt, Germany) was coated with 5 μg rSsEno/mL in PBS and at 4 °C (overnight). The plate was washed with washing buffer (0.1% Tween 20) and then blocked 1 h at room temperature with 5% dried skim milk in washing buffer. Dilutions of the serum samples (1:500 in blocking buffer) were added to each well and incubated at room temperature for 2 h. After washing, peroxidase-conjugated anti-mouse IgG (1/500) (Merck KGaA Darmstadt, Germany) was added and incubated at 37 °C for 1 h. After exhaustive washing, tetramethylbenzidine was added to reveal the antigen-antibody reactions (30 min at room temperature). The reaction was stopped by the addition of 50 μL/well 1M H_2_SO_4_, and the absorbance was read with an ELISA reader (Multiskan Ascent, Labsystem, Vantaa, Finland) at 450 nm.

### 2.8. Th1-Th17 Phenotipagem 

Spleens were aseptically removed and splenocytes were extracted. Viable splenocytes were adjusted to 1 × 10^7^ cells/mL in complete RPMI-1640 culture medium (Merck KGaA Darmstadt, Germany), which was supplemented with 2 mm l-glutamine, 100 U/mL penicillin, 100 μg/mL penicillin/streptomycin, and 10% fetal calf serum (RPMI complete). For study of Th1 and Th17 lymphocytes subpopulations, the following anti-mouse mAb were used: anti-CD16/CD32, anti-CD3-FITC, anti-CD4-APC, anti-IL-17-PE, anti-IFN-Ɣ-Percp, and respective isotype controls (all purchased from BD Biosciences, (Franklin Lakes, NJ, USA). Splenocytes were assessed for the frequency of Th1(IFN-Ɣ+), Th17 (IL-17+). Briefly, viable splenocytes were stained for the extracellular markers, then fixed and permeabilized using eBiosciences’ intracellular fixation (Thermo Scientific, Waltham, MA, USA) and permeabilization buffer set, and then the intracellular IFN-Ɣ and IL-17A were stained with a fluorescent respective marker. Intracellular cytokines were detected after in vitro stimulation with 10 µg/mL of rSsEno and Brefeldin A for intracellular retention of the induced cytokine. Events were acquired using a BD Accuri C6 flow cytometer (BD Biosciences) and analyzed with the flow cytometer’s proprietary software.

### 2.9. IFN-Ɣ, IL-4, and IL-17 Measurement in Supernatant of Splenocytes Culture

Splenocytes from immunized and non-immunized mice were cultured as previously described and stimulated with 10 µg/mL of rSsEno for 24 h. The levels of IFN-Ɣ, IL-4, and IL-17 after rSsEno stimulation were measured in the supernatant of splenocytes culture by Cytometric Bead Array (CBA) (BD Biosciences) according to the manufacturer’s instructions using a BD Accuri C6 flow cytometer (BD Biosciences).

### 2.10. Fungal Challenge and Infection Assessment

Either vaccinated or non-vaccinated mice were intraperitoneally inoculated with 10^6^ conidia of either wild type (WT) or toluene-adapted (Tadap) *S. schenckii* suspended in 100 µL of PBS or with an equal volume of PBS alone as control. To confirm the fungal cell count and viability of the inoculum, appropriately diluted samples of the conidia suspension were plated onto Mycosel agar plates and after seven days of incubation growing colonies were counted. At seventh day post-infection the mice were euthanized in CO_2_ chamber. The liver and spleen of each animal were removed to measure the relative organ weight and assess the systemic fungal load. The relative weight of livers and spleens was calculated by the following formula:

Related weight = organ weight (g)/body weight (kg).

To evaluate the fungal load, liver and spleen were macerated under sterile conditions and adequate dilutions of the macerate in PBS were cultured in duplicate, on Mycosel agar plates and the growing CFU were counted after three and six days. The final count was adjusted according to the dilution used.

### 2.11. Statistical Analysis

Statistical analysis was performed in GraphPad Prism ver. 6.01 (San Diego, CA, USA). A one-way analysis of variance (ANOVA) with Tukey comparisons test was used. The confidence interval was set at 95% for all tests. The significance level and *p*-values were shown as * (*p* < 0.05); ** (*p* < 0.01); *** (*p* < 0.001); **** (*p* < 0.0001).

## 3. Results

### 3.1. Production and Purification of rSsEno

Figure 1C,D show that the production and purification of rSsEno was effective. SDS-PAGE showed a unique band stained with Coomassie blue with the expected molecular mass of 47 kDa previously characterized as rSsEno [13]. In addition, a pooled anti-rSsEno serum obtained from mice immunized with Freund´s Adjuvants/rSsEno recognized specifically the rSsEno band in the immunoblotting while there were not detected non-specific binding in the control strip treated with a serum from non-immunized mice.

### 3.2. Post-Vaccination rSsEno-Antibody Response 

**Figure 2:**
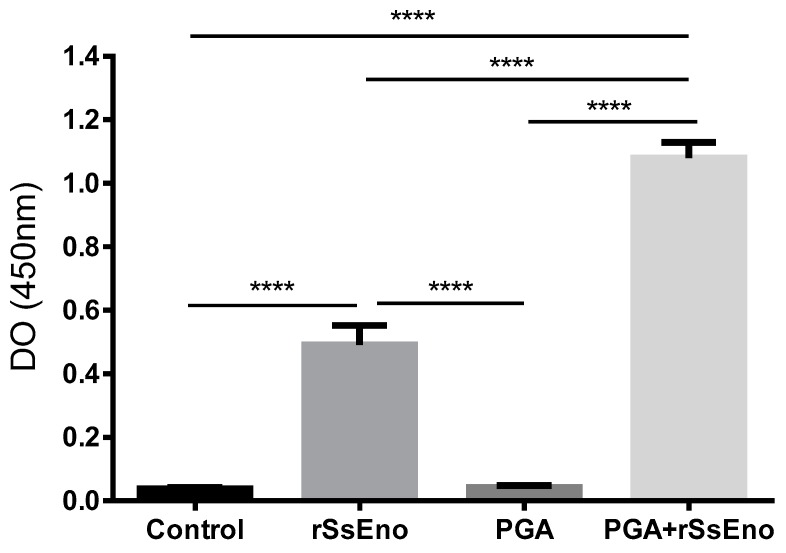
Immunization with PGA+rSsEno markedly enhanced the IgG antibody response to rSsEno. Balb/c mice were immunized (s.c.) twice with rSsEno, PGA, PGA+rSsEno, or PBS as negative control. Serum collected seven days after the second immunization was used to determine rSsEno-specific IgG antibody. **** (*p* < 0.0001).

The rSsEno-specific IgG antibody reaction after the second immunization with or without the PGA are displayed in Figure 2 as optical density (OD) measured at 450 nm. Immunization with PGA+rSsEno markedly enhanced the IgG antibody response to rSsEno compared to the response induced by the non-adjuvanted vaccine, seven days after the second immunization (*p* < 0.0001).

### 3.3. Th1 and Th17 Response

**Figure 3:**
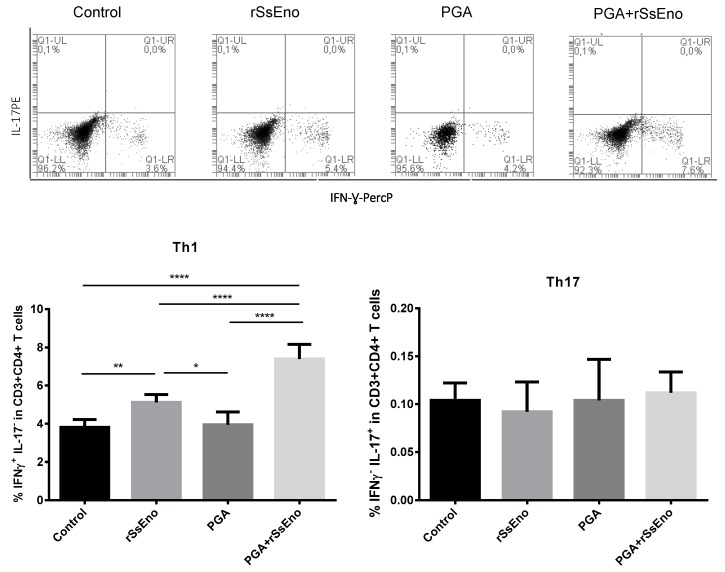
Response of Th1 (intracellular IFN-Ɣ+) and Th17 (intracellular IL-17A+) cells induced by immunization with the PGA+rSsEno. Balb/c mice were immunized (s.c.) twice with rSsEno, PGA, PGA+rSsEno, or PBS as negative control. Splenocytes of each animal were collected seven days after the second immunization and purified cells were stimulated in vitro with rSsEno and Brefeldin A. The frequency of Th1 and Th17 cells was detected using a BD Accuri C6 flow cytometer. Upper figure shows representative dot plots of Th1 and Th17 frequency collected from each group. * (*p* < 0.05); ** (*p* < 0.01); *** (*p* < 0.001): **** (*p* < 0.0001).

**Figure 4:**
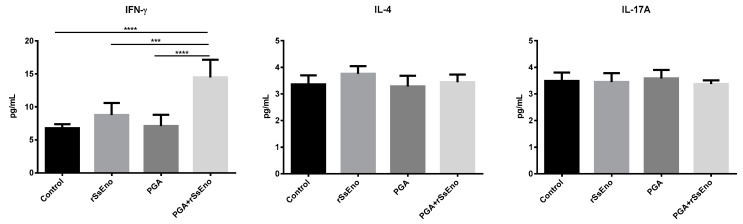
Cytokine profile in supernatant of splenocytes stimulated in vitro with rSsEno. Balb/c mice were immunized (s.c.) twice with rSsEno, PGA, PGA+rSsEno, or PBS as negative control. Splenocytes of each animal were collected seven days after the second immunization and purified cells were stimulated in vitro with rSsEno. Cytokine were quantified by cytometric bead array (CBA). * (*p* < 0.05); ** (*p* < 0.01); *** (*p* < 0.001): **** (*p* < 0.0001).

The response Th1 and Th17 are determinant in the immune response against *S. schenckii*. Mice vaccinated with rSsEno+PGA induced a significant response of Th1 lymphocytes after in vitro stimulation with rSsEno compared with the other groups (*p* < 0.0001). However, the response of Th17 lymphocytes was not modified in any experimental group (Figure 3). A similar response was observed when the concentration of IFN-Ɣ, IL-4, and IL-17A were measured by CBA. A high production of IFN-Ɣ, belonging to the Th1 pattern was detected in the group vaccinated with rSsEno+PGA compared with non-adjuvanted *rSsEno* vaccination (*p* < 0.001) and with the control groups (*p* < 0.0001) (Figure 4). 

### 3.4. Fungal Challenge and Infection Assessment

**Figure 5:**
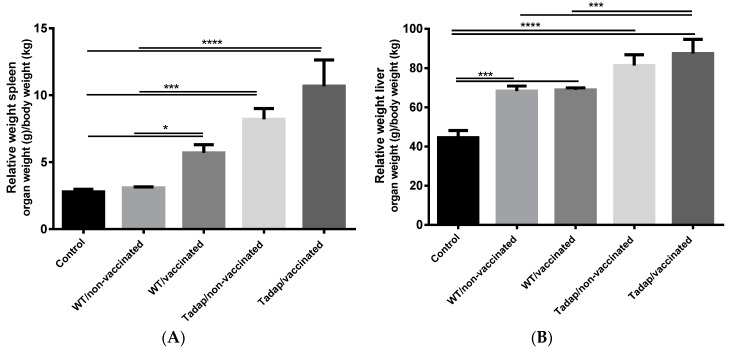
Relative (**A**) spleen and (**B**) liver weight. Balb/c mice were injected (s.c.) twice with PGA+rSsEno (vaccinated) or PBS (non-vaccinated). Seven days after the second dose of administration of PGA+rSsEno or PBS, mice were infected intraperitoneally with 1 × 10^6^ conidia of either wild type (WT) or toluene adapted *S. schenckii* (Tadap). The spleen and the liver of each animal were collected seven days after the second immunization and the relative weight (organ weight/animal weight) was measured. * (*p* < 0.05); *** (*p* < 0.001); **** (*p* < 0.0001).

The aim of this study was to investigate if the immune response induced by rSsEno adjuvanted with PGA was able to protect against a challenge with either WT or Tadap *S. schenckii.* We analyzed the spleen and the liver as representative organs to evaluate the fungal load. All the infected mice developed hepatomegaly that was observed by measuring the relative weight of the liver. However, splenomegaly was also observed in mice vaccinated and infected. Those animals vaccinated and infected with highly virulent toluene-adapted *S. schenckii* developed the greatest hepato- and splenomegaly compared with the control group (*p* < 0.0001) (Figure 5). 

**Figure 6:**
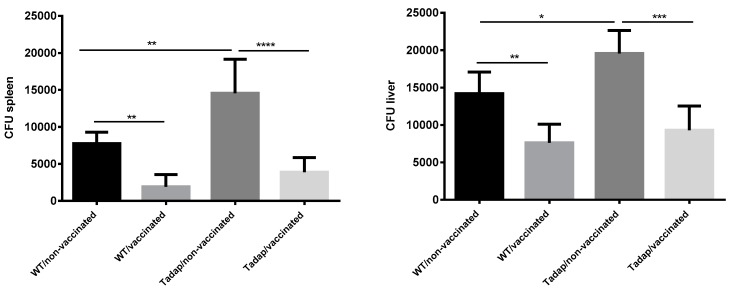
Vaccination with PGA+rSsEno was able to reduce the fungal burden in spleen and liver of mice infected with either wild type (WT) or toluene adapted *S. schenckii* (Tadap). Balb/c mice were injected (s.c.) twice with PGA+rSsEno (vaccinated) or PBS (non-vaccinated). One week after the boost, mice were i.p. challenged with *S. schenckii* and seven days after infection the protection was assessed by the number of CFUs recovered from the spleen and liver. * (*p* < 0.05); ** (*p* < 0.01); *** (*p* < 0.001); **** (*p* < 0.0001).

The protective effect induced by the adjuvanted vaccine after the fungal challenge is shown in Figure 6. The immune response induced by PGA+ rSsEno was able to reduce the fungal burden in spleen and liver of mice infected with either WT or Tadap *S. schenckii* in a similar way. However, owing to the higher virulence of Tadap *S. schenckii*, this finding suggests that PGA+rSsEno can be effective against fungus with different levels of virulence. 

## 4. Discussion

Recent advances in the understanding of relevant immunological mechanisms against pathogenic fungi favor the development of prophylactic and therapeutic antifungal vaccines [23,24,25]. In contrast to classical antifungal medications, vaccines can be administered to large populations with low potential risks or side-effects [26]. Currently, there is a growing tendency to develop subunit vaccines, based in well-defined microbial components, in order to increase vaccine safety [27]. Unfortunately, pure antigens are poorly immunogenic, and they should be formulated with adjuvants to improve the vaccine efficacy [14,28].

Several non-toxic polymer adjuvants are being used for sustained delivery of protein subunit vaccines [29,30]. Polymeric adjuvants act through slow release of the antigen for the selective targeting to antigen presenting cells, promoting different signaling pathways including, activation of toll-like receptor(s) and inflammasome pathway or directly interacting with B cells. Polymer–antigen complex can be phagocytosed, and the antigen effectively presented to naive T cells via major histocompatibility complex (MHC) molecules [31]. 

Recently, we compared AH with the polymeric adjuvant PGA in a vaccine candidate against *S. schenckii* containing proteins extracted from the fungal cell wall. PGA induced a protective immune response against both *S. schenckii* and *S. brasiliensis* with lesser local toxicity than AH in vaccinated mice [21]. In other recent work, we developed recombinant enolase that was a key antigen in SsCWPs and it was evaluated in a vaccine formulated with PGA as an adjuvant. Again, protective immune response in vaccinated mice after a challenge with *S. brasiliensis* was observed with a survival rate above 90% after 45 days of intravenous fungal challenge [22]. 

Here, we evaluated the protective effect of PGA formulated with rSsEno against a highly virulent *S. schenckii.* The enhanced virulence was acquired after fungal exposure to 0.1% of toluene, which induced adaptive changes previously described, including enhanced melanosome formation and stronger antioxidant mechanism compared with the wild type strain [7]. 

Before the evaluation of the protective effect of vaccination, the immunogenicity of the vaccine after two subcutaneous doses of PGA+rSsEno was evaluated. In the aforementioned work [22], we evaluated the immunogenicity and efficacy of this vaccine candidate using three subcutaneous administrations and PGA 10%. However, here the immunogenicity of the vaccine was evaluated after two doses and PGA 5%, as we used in another vaccine candidate with SsCWPs [21]. As expected, the formulation of PGA+rSsEno induced elevated production of specific IgG antibodies and a strong Th1 response after in vitro stimulation of splenocytes with rSsEno. However, we did not observe a significant Th17 response analyzed by both intracellular and released IL-17A.

Several studies revealed that Th1 response plays a decisive role in the defense against *S. schenckii* infection [32,33,34,35]. In this sense, activation of Th1 lymphocytes is becoming an interesting immunomodulatory strategy against sporotrichosis. Flores-García et al. (2015) reported that treatment with recombinant murine IL-12 (rmIL-12) promotes Th1 immunity and clinical improvement in an experimental sporotrichosis gerbil model [36]. In other study, Batista-Duharte et al. (2016) evaluated the therapeutic effect of adjuvant Finlay cochleates 3 (AFCo3), a cochleate containing purified and non-toxic LPS derived from *Neisseria meningitidis B* as vehicle of Amphotericin B (AmB) to evaluate the combined effect of immunomodulation induced by AFCo3 and the antifungal effect in a murine model of *S. schenckii* infection. AFCo3 stimulated a strong Th1- and Th17 response associated with the antifungal effect of AmB, which significantly improved the fungal clearance [37]. Regarding vaccination, studies suggest that Th1 response is associated with anti-sporothrix vaccine protection [20,21,22,38,39] and the activation of dendritic cells by fungal wall proteins seems to be important in theTh1 bias [34]. However, the role of Th17 response to prevent sporotrichosis is controversial. Some studies reveal that anti-sporothrix vaccination can induce Th17- combined with Th1 response [20,38,39,40]. However, two studies using PGA as an adjuvant with either SsCWPs [21] or rSsEno [22], showed that Th1 response is sufficient to achieve protection against a challenge with *S. schenckii* or *S. brasiliensis*. In the previous comparative study, using SsCWPs as antigen with either AH or PGA as adjuvant revealed that the adjuvant is the main component that drives the quality of the immune response since AH induced a balanced Th1/Th2/Th17 response while PGA induced a Th1 response [21]. Interestingly, the reduction of fungal load in both groups was very similar despite having different overall Th cell pattern. 

In this work, we observed that the anti- rSsEno Th1 response induced by PGA confers protection against Tadap *S. schenckii* after an intraperitoneal fungal challenge. Previously, we showed that infected mice with Tadap *S. schenckii* produced high levels of IFN-Ɣ and, to a lesser extent, IL-17 [7]. This finding reinforces the criterion of the role of Th1-mediated response for protection against *S. schenckii* infection. Here, we observed significant production of IFN-Ɣ after in vitro stimulation with rSsEno, compared to non-vaccinated mice, although the cytokine production was low in general. However, unlike infection where multiple lymphocyte clones are activated, vaccination stimulates specific clones, but they are sufficient in the booster phase to prevent infection. In this way, the role of vaccination is the expansion of specific lymphocytes to maintain a basal level of memory cell able to rapidly respond against microbial infection [41].

These results are important as part of the studies of the efficacy of the recombinant enolase-Montanide™ PetGel A vaccine, due to the environment can exert a direct influence on fungal virulence [4,5]. Ideally, a prophylactic vaccine should be able to prevent infections caused by microorganisms with different levels of virulence since imperfect vaccination can enhance the transmission of highly virulent pathogens [42]. In this sense, the choice of potent and safe adjuvants driving effective immunological mechanisms against the target microorganism is one of the most important factors for vaccine efficacy [28]. However, keeping in mind that besides the antigen and the adjuvant mode of action, vaccine efficacy and toxicity are strongly influenced by the genetic background of the host [43,44], additional studies in different mouse strains and non-rodent species will be performed. 

## 5. Conclusions

In this work, we provide evidence that vaccination with a recombinant enolase-based vaccine formulated with PGA as an adjuvant promotes a protective immune response in mice, against a highly virulent *S. schenckii* by toluene exposure. To the best of our knowledge, this is the first experimental approach to assess the role of vaccination in providing protective immunity against pathogenic fungus with enhanced virulence by exposure to environmental contaminants. Further studies are necessary in order to evaluate the memory response, including other mouse strains and using a model of subcutaneous infection.

## Figures and Tables

**Table 1:** General properties of Montanide™ GEL adjuvants.

	Description
Composition	Gel particles of sodium polyacrylate in water.
Particle size	90% of the particles are smaller than 1.2 μm in diameter.
Stability	Highly stable at room temperature.
Mechanisms of action	Depot effect with slow release of antigens, due to polymer adsorption properties. Improves the recruitment and activation of the innate immune system cells and inducement of specific immune response.
Vaccine preparation	Montanide™ GEL adjuvants are ready-to-use adjuvants that can be combined with a wide range of antigens by gentle mixing.
Routes of administration	Parenteral and mucosal administration.
Uses	Montanide™ GEL adjuvants are recommended for a wide variety of livestock species and for pets and horses. They can be formulated with a wide range of antigens.
Safety	Montanide™ adjuvants and their components have been considered as safe by the Committee for Veterinary Medical Products (CVMP) for use in immunological products. They are included in Part I of the Annex of the European Council Regulation n° 37/2010/EU as substances needing no further MRL studies, in the Out of Scope list (EMACVMP-519714-2009), and included in already registered veterinary commercial products.
